# Usefulness of presepsin in the diagnosis of sepsis in patients with or without acute kidney injury

**DOI:** 10.1186/1471-2253-14-88

**Published:** 2014-10-04

**Authors:** Yoshihiko Nakamura, Hiroyasu Ishikura, Takeshi Nishida, Yasumasa Kawano, Rie Yuge, Reiko Ichiki, Akira Murai

**Affiliations:** Department of Emergency and Critical Care Medicine, Faculty of Medicine, Fukuoka University, 7-45-1 Nanakuma, Jonan-ku, Fukuoka, 814-0180 Japan

**Keywords:** Presepsin, Acute kidney injury, RIFLE criteria, Sepsis, Diagnosis

## Abstract

**Background:**

Presepsin is useful for differentiating sepsis from non-infection related systemic inflammatory response syndrome. However, there are no studies investigating the usefulness of presepsin in diagnosing sepsis involving patients with acute kidney injury (AKI). The purpose of this study is to determine levels of blood presepsin in patients with or without sepsis and among non-AKI patients or patients with different degrees of AKI severity.

**Methods:**

This is a single center retrospective study. 247 patients admitted to the ICU between June 2010 and October 2012 were analyzed for their presepsin levels. We classified the patients into non-AKI and AKI according to the RIFLE (Risk, Injury, Failure, and Loss of kidney function and End-stage kidney disease or simply Loss and ESKD) criteria. We then sub-classified the patients in each group into either non-sepsis or sepsis sub-group and analyzed the accuracy of diagnosing sepsis based on their levels of presepsin.

**Results:**

The number of patients for each group was: non-AKI, 112; under AKI: Risk, 50; Injury, 36; Failure, 42; Loss and ESKD, 7. The levels of presepsin in sepsis groups were significantly higher than that in the non-sepsis group among the non-AKI, Risk and Injury patients (p < 0.0001, p < 0.01, p < 0.01, respectively). However, no significant difference in the level of presepsin between non-sepsis and sepsis groups among patients with Failure. In the receiver operating characteristic (ROC) analysis, the area under the curve (AUC) was 0.784 in the non-AKI group and 0.698 in the AKI comprising Risk, Injury and Failure groups. AUC value for non-AKI was not significantly different from that of AKI (p = 0.200). When 670 pg/mL was used as the cutoff value for presepsin, sensitivity and specificity were 70.3% and 81.3%, respectively. When 864 pg/mL was used as the cutoff value for presepsin, sensitivity and specificity were 71.4% and 63.8%, respectively.

**Conclusions:**

Presepsin level can be a reliable indicator of sepsis not only among non-AKI patients but also patients with less severe forms of AKI. However, it may not be a reliable indicator of sepsis in patients with a more advanced form of AKI.

## Background

Since the definition of systemic inflammatory response syndrome (SIRS) was proposed in 1991, several clinical trials on sepsis diagnosis and treatment have been conducted using the definition of sepsis given by the American College of Chest Physicians/Society of Critical Care Medicine (ACCP/SCCM) [1]. Many studies have reported that early treatment of sepsis using appropriate antibiotics improved the prognosis and increased the survival rate in severe sepsis or septic shock patients [2–4]. Various biomarkers have been studied for diagnosing sepsis [5]. Currently, procalcitonin (PCT) is used as a marker to diagnose sepsis or severe sepsis. In comparison to other markers that have traditionally been reported, PCT gives a high rate of specificity for sepsis diagnosis [6]. However, the concentration of PCT in the human blood is elevated in various conditions, such as in severe trauma, surgical invasive procedures, and critical burn injury, which leads to SIRS. It is also necessary to be aware of false-positive results [7]. Therefore, more reliable biomarkers for the diagnosis of sepsis are needed. Another marker is interleukin-6 (IL-6), which may be detectable in the early stages of infection and bacteremia [8, 9]. Recently Endo et al. [10] reported that presepsin is a highly specific marker for diagnosis of bacterial infections in comparison to other sepsis markers (PCT, IL-6). However, the presepsin levels above the cutoff value in patients with chronic renal failure must be interpreted with caution. Additionally, there are no studies investigating the usefulness of presepsin for assessing patient with acute kidney injury (AKI). In this study we attempted to clarify the diagnostic accuracy of sepsis using the presepsin level according to AKI severity of the patients.

## Methods

**Figure 1:**
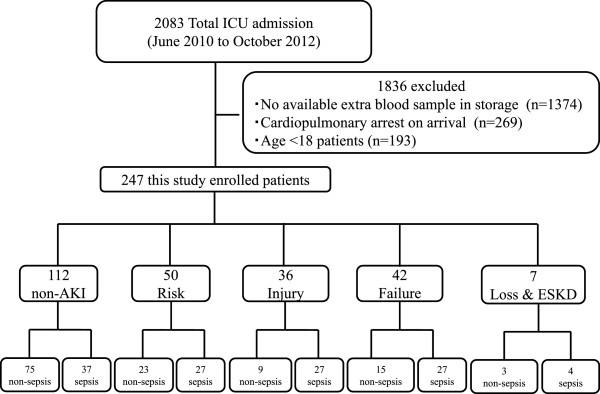
**Patients enrollment, exclusion, and classification.** AKI, acute kidney injury.

This study was conducted as a single center retrospective study. In our hospital, patient’s extra blood samples were stored for research purposes and informed consent from patients taken. Whole blood was collected with EDTA-2 K as an anticoagulant using a conventional blood collection tube (TERUMO, Japan). Whenever extra blood sample is available, the blood is centrifuged at 1400 g for 5 min and the serum kept at -70°C for future use. In this present study, we enrolled all qualified patients with available stored serum samples. The study protocol was approved by the institutional review board of Fukuoka University Hospital according to the Declaration of Helsinki. The samples were from patients’ blood collected 24 hr upon admission to the ICU of Fukuoka University Hospital between June 2010 and October 2012. There were 247 patients with stored samples and we included all for presepsin measurement. We grouped the patients into non-AKI or AKI according to the RIFLE (Risk, Injury, Failure, Loss of kidney function and End-stage kidney disease: Loss and ESKD) criteria [11]. We then classified patients in each group as either sepsis or non-sepsis and analyzed the accuracy of diagnosing sepsis based on the levels of presepsin. Sepsis diagnosis (include severe sepsis and septic shock) was defined by ACCP/SCCM Consensus Conference Committee [1]. Patients’ classification, enrollment into the study and exclusion from the study are shown Figure 1.

### Presepsin measurement

The frozen serum was allowed to melt to become liquid at room temperature before presepsin testing. Presepsin concentration in blood was measured with a compact automated immunoanalyzer, PATHFAST^®^, based on a chemiluminescent enzyme immunoassay (CLEIA) (Mitsubishi Chemical Medience, Japan) [12, 13].

### Classification of acute kidney injury

Oliguria and RIFLE were the most frequently used criteria to define AKI. In particular, RIFLE criteria were formulated to make a clearer classification of AKI among patients having renal problems [14]. Therefore, we used RIFLE criteria for AKI diagnosis in this study.

The patient’s degree of AKI according to the RIFLE criteria (Table 1) [11] is determined after the urine output for the first 24 hr upon admission has been measured. RIFLE criteria involve measurement of blood creatinine (Cr) and monitoring of 24 hr urine output to classify patient’s state of renal injury. In this study, baseline Cr was determined based on patient’s available record prior to ICU admission or, if such record was not available, the lowest level of Cr during the course of ICU admission. To determine the AKI status of a patient, we used urine GFR criteria and urine output criteria or, if the two differs, we used the one that gives a more severe AKI classification.

**Table 1:** **RIFLE criteria** ^**11)**^

RIFLE classification	GFR criteria	Urine output criteria
Risk	Increased serum creatinine × 1.5 or GFR decrease > 25%	Urine output < 0.5 ml/kg/h × 6 hr
Injury	Increased serum creatinine × 2 or GFR decrease > 50%	Urine output < 0.5 ml/kg/h × 12 hr
Failure	Increased serum creatinine × 3 or GFR decrease > 75%, serum creatinine ≧ 4 mg/dL (acute rise > 0.5 mg/dL)	Urine output < 0.3 ml/kg/h × 24 hr or Anuria × 12 hr
Loss	Persistent ARF = complete loss of kidney function > 4 weeks	
ESKD	End Stage Kidney Disease (>3 months)	

The criteria that lead to the worst possible classification should be used. RIFLE, Risk of renal dysfunction, Injury to the kidney, Failure of kidney function, Loss of kidney function and End-stage kidney disease; GFR, Glomerular Filtration Rate; ARF Acute Renal Failure.

### Estimated glomerular filtration rate (eGFR)

We calculated eGFR by using a Japanese equation [15].


### Statistical analysis

Continuous variables are presented as median and range. Groups were compared by Wilcoxon test. Analysis of the values of area under curve (AUC) of receiver operating characteristic (ROC) curves was performed to determine the significance of presepsin levels in diagnosing sepsis. The Youden index was used to identify the cutoff values for presepsin levels that may have diagnostic significance. Correlations between presepsin levels and Cr or eGFR were evaluated by the Spearman’s rank test. All statistical analyses in this study were performed using JMP^®^ version 10 and MedCalc^®^ version 13. P value less than 0.05 was considered statistically significant.

## Results

**Figure 2:**
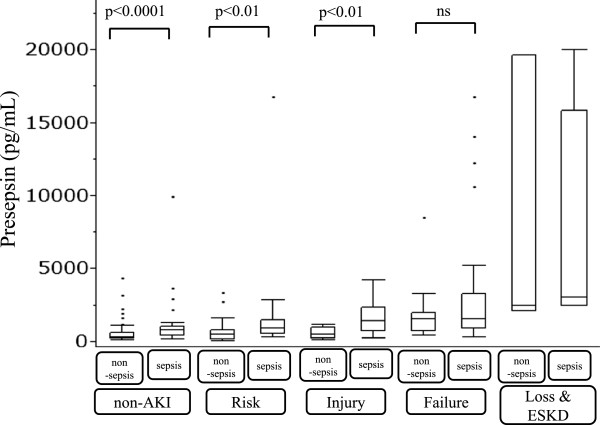
**The comparison of the presepsin levels between non-sepsis and sepsis among non-AKI patients and patients with varying degrees of AKI.** Significant difference in presepsin levels between non-sepsis and sepsis patients were observed among non-AKI and AKI under Risk and Injury groups. However, no significant deference was observed in AKI Failure group. No statistical comparison on the Loss & ESKD group due to the small number of patients (n = 7) available and the abnormally high levels of presepsin detected in this group.

**Figure 3:**
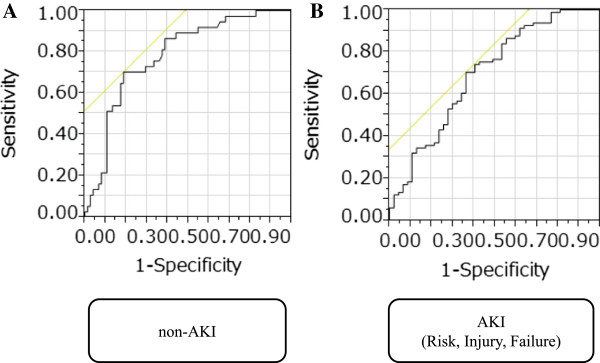
**Receiver operating characteristic (ROC) curves of presepsin in patients with non-sepsis and sepsis. A**. The ROC for non-AKI group (AUC = 0.784). **B**. The ROC for AKI comprising the Risk, Injury and Failure groups (AUC = 0.698).

There were 247 patients (141 men, 106 women) whose median age was 70 (range: 18–96) years old enrolled in this study. The underlying diseases of patients are shown in Table 2. The number of sepsis patients is 122 (sepsis, n = 30; severe sepsis, n = 40; septic shock, n = 52). The results of the bacteriological examination in sepsis patients are shown in Table 3. One hundred and twelve patients were non-AKI and we classified the patients with AKI according to the RIFLE criteria. Fifty patients were classified Risk, 36 were Injury, 42 were Failure, and 7 were Loss and ESKD. The median level of presepsin was 300 pg/mL (range: 86–4374) in the non-AKI with non-sepsis group (n = 75), 831 pg/mL (range: 187–9960) in the non-AKI with sepsis group (n = 37); 467 pg/mL (range: 71–3361) in the Risk non-sepsis group (n = 23), 924 pg/mL (range: 290–16759) in the sepsis group (n = 27); 517 pg/mL (range: 144–1197) in the Injury non-sepsis group (n = 9), 1451 pg/mL (range: 237–4200) in the sepsis group (n = 27). The presepsin levels in the sepsis groups were significantly higher than the non-sepsis groups (p < 0.0001, p < 0.01, p < 0.01, respectively). The median level of presepsin was 1535 pg/mL (range: 454–8516) in the Failure non-sepsis group (n = 15) and 1523 pg/mL (range: 293–16764) in the sepsis group (n = 27). There was no significant differences in presepsin levels between non-sepsis group and sepsis group (p = 0.300) in the Failure group (Figure 2). As for the accuracy of diagnosing sepsis based on the level of presepsin in the ROC analysis, the AUC was 0.784 (95% CI: 0.683-0.860) in the non-AKI group and 0.698 (95% CI: 0.593-0.786) in the Risk, Injury and Failure groups combined (Figure 3). The AUC value for non-AKI group was not significantly different from that of AKI under Risk, Injury and Failure groups combined (p = 0.200). The Loss & ESKD under AKI was excluded due to a very small number of patients (n = 7) for statistical analysis, and also due to abnormally high values of presepsin detected in the samples (see Table 4). When 670 pg/mL was used as a cutoff value for presepsin, sensitivity and specificity were 70.4%, and 81.3%, respectively; and when 864 pg/mL was used as a cutoff value for presepsin, sensitivity and specificity were 71.3%, and 63.8%, respectively.

**Table 2:** **The background of patients**

Non-sepsis	Trauma	20	125
	Metabolism, Endocrine, Allergic disease	16	
	Circulatory disease	11	
	Stroke, Epilepsy	10	
	Pneumonia	8	
	Respiratory disease	7	
	Pancreatitis	6	
	Heat stroke	5	
	Bone and soft tissue infection	5	
	Burn	5	
	Liver disease	4	
	Abdominal cavity or intestinal infection	3	
	Drug poisoning	3	
	Urinary tract infection	3	
	Renal disease	2	
	Others	17	
Sepsis	Pneumonia	40	122
	Abdominal cavity or intestinal infection	37	
	Bone and soft tissue infection	20	
	Urinary tract infection	7	
	Focus unknown	6	
	Others	12

**Table 3:** **Results of bacteriological examination in sepsis patients**

Etiologic agent*	***n***
Not detected or not examined	36
Gram negative rods	35
Gram positive coccus	26
Gram positive coccus and Gram negative bacillus	16
Gram positive coccus and Fungus	4
Fungus	2
Gram positive coccus and Gram negative bacillus and Fungus	1
Mycobacterium tuberculosis	1
Virus+	1
**Total**	**122**

*Microbiological test result of samples taken from the presumed site of infection or blood culture.

+Cytomegalovirus infection (diagnosed by Antigenemia method; C7HRP).

**Table 4:** **Loss of the kidney function and End-stage renal disease character of patients**

Patient* no.	Sepsis	Presepsin (pg/mL)	The background of patients
1	-	2457	Renal disease
2	-	2134	Others
3	-	19633	Urinary tract infection
4	+	20000	Others
5	+	3424	Pneumonia
6	+	2450	Pneumonia
7	+	2632	Pneumonia

*Patient’s age ranges from 63 to 80 years old (3 females and 4 males).

**Figure 4:**
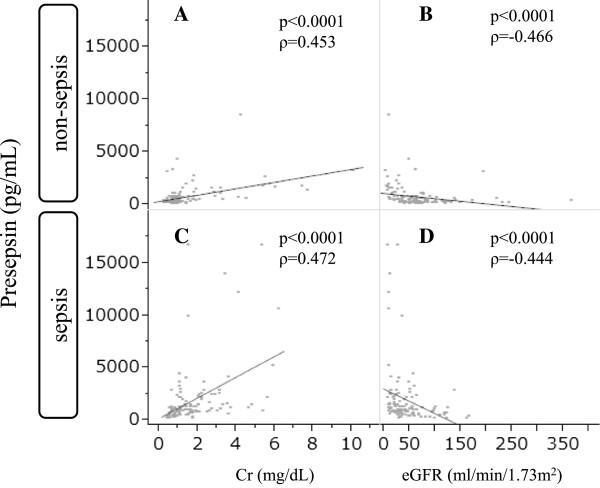
**Spearman’s rank correlation between presepsin levels and creatinine (Cr) or between presepsin and estimated glomerular filtration rate (eGFR).** The correlations between presepsin levels and Cr (ρ = 0.453, p < 0.0001) **(A)** or eGFR (ρ = -0.466, p < 0.0001) **(B)** in non-sepsis patients. The correlations between presepsin levels and Cr (ρ = 0.472, p < 0.0001) **(C)** or eGFR (ρ = -0.444, p < 0.0001) **(D)** among patients with sepsis.

To determine how renal function affects presepsin level, correlation between presepsin and Cr or eGFR was evaluated using Spearman’s rank test. The results showed that positive correlation between presepsin and Cr, and negative correlation between presepsin and eGFR are significantly similar between non-sepsis and sepsis groups (Figure 4). This finding, in addition to the abnormally high presepsin levels among patients with end-stage AKI, suggests that the kidney is the main organ responsible in clearing blood of presepsin.

## Discussion

Presepsin is a 13-kDa protein that is a fragment of CD14 with truncated N-terminal, the receptor for lipopolysaccharide (LPS)/LPS binding protein (LBP) complexes [16, 17]. An *in vivo* study using rabbit sepsis models showed that presepsin level did not increase in the LPS-induced sepsis model whereas elevation of presepsin level was observed in a cecal ligation and puncture (CLP) sepsis model. It was speculated that the infectious stimulus led to the elevation of presepsin level. One of the production mechanisms of presepsin is related to the phagocytosis process and cleavage of membrane CD14 with lysosomal enzymes of granulocytes in an *in vitro* study using rabbit peritoneal leukocyte [18]. The biological function of presepsin, however, remains unknown [17]. Recently, presepsin is considered a novel marker for the diagnosis of sepsis that has been shown to increase in blood in the early stages of sepsis [19]. A multicenter prospective study by Endo et al. [10] showed that presepsin is a highly specific marker for diagnosis of bacterial infections in comparison to other sepsis markers (PCT, IL-6).

In the present study, we observed that presepsin was significantly increased in sepsis patients with non-AKI and patients with milder forms of AKI (Risk and Injury) compared to non-sepsis groups. Furthermore, the median value of presepsin increased with increasing severity of AKI both in the non-sepsis and sepsis groups. The optimal cutoff value of presepsin in non-AKI group was 670 pg/mL (a result similar to that by Endo et al. [10]) while the optimal cutoff value of presepsin in the AKI group (Risk, Injury and Failure groups combined) was significantly higher at 864 pg/mL. However, when the Failure group was separately analyzed, the result is that presepsin levels among patients with sepsis are not significantly different than those with non-sepsis in this particular group of AKI patients. Despite the limited number of patients in Loss and ESKD group, the levels of presepsin were consistently and abnormally high in all patients, whether they have sepsis or not (see Table 4). It is therefore suggested that further study is needed to establish cutoff value of presepsin for each level of AKI severity to diagnose sepsis in these groups of patients.

As to how our body clears the blood of presepsin remains unknown. In this study, our data show that there is moderate positive correlation between presepsin and Cr, and also moderate negative correlation between presepsin and eGFR in non-sepsis groups that were significantly similar to that of the sepsis groups. These results suggest that kidney is the major organ responsible in eliminating presepsin from blood. Since renal function could be considered a major determinant of presepsin level, it is therefore logical that different thresholds of presepsin levels (according to the status of renal function) be applied to diagnose sepsis.

Many studies have reported that early treatment of sepsis using appropriate antibiotics improved the prognosis and increased the survival rate in severe sepsis or septic shock patients [2–4]. Moreover, patients with AKI are susceptible to many complications such as those that may be caused by sepsis; and conversely, the most common cause of AKI in critically ill patients is sepsis [20], thus making early diagnosis and treatment of sepsis very important.

Blood culture is frequently used as the “gold standard” diagnostic method for diagnosing sepsis. However, blood culture usually takes 3 to 7 days to obtain the results and frequently yields low true positive results [21]. Therefore, in general practice, the decision to treat patient for sepsis is based on the doctor’s own experience (empiric therapy). Any quick tests that indicate sepsis will certainly improve the physician’s changes of making the right diagnosis of sepsis. Presepsin testing could be one of these tests. Presepsin levels in blood are known to increase in the first 6 hr after the onset of sepsis. These changes in concentration occurred on a much faster time scale than those observed for PCT or CRP [22]. The PATHFAST® presepsin assay reveals its result within 17 min. This is faster than the other methods of detecting presepsin by ELISAs [22, 23]. The PATHFAST® presepsin assay can be performed using whole blood. Whole blood samples are suitable for use in the emergency room, ICU, and the surgical operation room, thus making this present study on presepsin more meaningful.

Some limitations in our study deserve consideration. First, this retrospective single center study only involved a small number of samples; second, we did not compare presepsin to other promising biomarkers of bacterial infection that have been recently proposed, such as PCT and IL-6; third, we did not consider the effect of cellular immunity status of the patients (such as patients under chemotherapy, steroids treatment, etc.) on presepsin level; and fourth, bacterial culture was not considered in the diagnosis of sepsis. Further studies are therefore needed to address the limitations of this study cited above.

## Conclusions

Blood presepsin level can be a reliable indicator of sepsis not only among non-AKI patients but also patients with less severe forms of AKI. However, it may not be a reliable indicator of sepsis in patients with a more advanced form of AKI, such as those classified under RIFLE criteria as having failure of kidney function, loss of kidney function and end-stage kidney disease.

## References

[1] Bone RC, Balk RA, Cerra FB, Dellinger RP, Fein AM, Knaus WA, Schein RM, Sibbald WJ (1992). Definitions for sepsis and organ failure and guidelines for the use of innovative therapies in sepsis. The ACCP/SCCM consensus conference committee. American college of chest physicians/society of critical care medicine. Chest.

[2] Battleman DS, Callahan M, Thaler HT (2002). Rapid antibiotic delivery and appropriate antibiotic selection reduce length of hospital stay of patients with community-acquired pneumonia: link between quality of care and resource utilization. Arch Intern Med.

[3] Kumar A, Roberts D, Wood KE, Light B, Parrillo JE, Sharma S, Suppes R, Feinstein D, Zanotti S, Taiberg L, Gurka D, Kumar A, Cheang M (2006). Duration of hypotension before initiation of effective antimicrobial therapy is the critical determinant of survival in human septic shock. Crit Care Med.

[4] Rivers E, Nguyen B, Havstad S, Ressler J, Muzzin A, Knoblich B, Peterson E, Tomlanovich M, Early Goal-Directed Therapy Collaborative G (2001). Early goal-directed therapy in the treatment of severe sepsis and septic shock. N Engl J Med.

[5] Bhatia BD, Basu S (2007). Newer diagnostic tests for bacterial diseases. Indian J Pediatr.

[6] Herzum I, Renz H (2008). Inflammatory markers in SIRS, sepsis and septic shock. Curr Med Chem.

[7] Christ-Crain M, Muller B (2005). Procalcitonin in bacterial infections–hype, hope, more or less?. Swiss Med Wkly.

[8] Oda S, Hirasawa H, Shiga H, Nakanishi K, Matsuda K, Nakamua M (2005). Sequential measurement of IL-6 blood levels in patients with systemic inflammatory response syndrome (SIRS)/sepsis. Cytokine.

[9] Abe R, Oda S, Sadahiro T, Nakamura M, Hirayama Y, Tateishi Y, Shinozaki K, Hirasawa H (2010). Gram-negative bacteremia induces greater magnitude of inflammatory response than gram-positive bacteremia. Critical care (London, England).

[10] Endo S, Suzuki Y, Takahashi G, Shozushima T, Ishikura H, Murai A, Nishida T, Irie Y, Miura M, Iguchi H, Fukui Y, Tanaka K, Nojima T, Okamura Y (2012). Usefulness of presepsin in the diagnosis of sepsis in a multicenter prospective study. J Infec Chemother Official J Japan Soc Chemotherap.

[11] Bellomo R, Ronco C, Kellum JA, Mehta RL, Palevsky P, Acute Dialysis Quality Initiative w (2004). Acute renal failure - definition, outcome measures, animal models, fluid therapy and information technology needs: the second international consensus conference of the acute dialysis quality initiative (ADQI) group. Critical care (London, England).

[12] Kurihara T, Yanagida A, Yokoi H, Koyata A, Matsuya T, Ogawa J, Okamura Y, Miyamoto D (2008). Evaluation of cardiac assays on a benchtop chemiluminescent enzyme immunoassay analyzer, PATHFAST. Anal Biochem.

[13] Okamura Y, Yokoi H (2011). Development of a point-of-care assay system for measurement of presepsin (sCD14-ST). Clinica Chim Acta Int J Clinical Chem.

[14] Ricci Z, Ronco C, D’Amico G, De Felice R, Rossi S, Bolgan I, Bonello M, Zamperetti N, Petras D, Salvatori G, Dan M, Piccinni P (2006). Practice patterns in the management of acute renal failure in the critically ill patient: an international survey. Nephrol dialysis Transplant Official Publ European Dialysis Transpl Assoc European Renal Assoc.

[15] Matsuo S, Imai E, Horio M, Yasuda Y, Tomita K, Nitta K, Yamagata K, Tomino Y, Yokoyama H, Hishida A, Collaborators developing the Japanese equation for estimated GFR (2009). Revised equations for estimated GFR from serum creatinine in Japan. Am J Kidney Dis Official J Nat Kidney Found.

[16] Furusako SSK (2008). Methods for detecting human low molecular weight CD14. United States Patent.

[17] Furusako SSK, Hirose J (2009). Soluble CD14 antigen. United States Patent.

[18] Naito K (2011). Method for evaluation of function of phagocyte. United States Patent.

[19] Shozushima T, Takahashi G, Matsumoto N, Kojika M, Okamura Y, Endo S (2011). Usefulness of presepsin (sCD14-ST) measurements as a marker for the diagnosis and severity of sepsis that satisfied diagnostic criteria of systemic inflammatory response syndrome. J Infect Chemotherap Official J Japan Soc Chemotherap.

[20] Murugan R, Kellum JA (2011). Acute kidney injury: what’s the prognosis?. Nat Rev Nephrol.

[21] Rangel-Frausto MS, Pittet D, Costigan M, Hwang T, Davis CS, Wenzel RP (1995). The natural history of the systemic inflammatory response syndrome (SIRS). A prospective study. JAMA J Am Med Assoc.

[22] Yaegashi Y, Shirakawa K, Sato N, Suzuki Y, Kojika M, Imai S, Takahashi G, Miyata M, Furusako S, Endo S (2005). Evaluation of a newly identified soluble CD14 subtype as a marker for sepsis. J Infect Chemotherap Official J Japan Soc Chemotherap.

[23] Shirakawa K, Naitou K, Hirose J, Takahashi T, Furusako S (2011). Presepsin (sCD14-ST): development and evaluation of one-step ELISA with a new standard that is similar to the form of presepsin in septic patients. Clinical Chem Lab Med CCLM / FESCC.

[CR24] The pre-publication history for this paper can be accessed here:http://www.biomedcentral.com/1471-2253/14/88/prepub

